# Corticoïdes et culture: un cas d'épisode psychotique aigu cortico-induit

**DOI:** 10.11604/pamj.2019.33.25.18207

**Published:** 2019-05-15

**Authors:** Mahmoud Amine Laffinti, Jalal El Ouadoudi, Hicham Guennouni Hassani, Rachid Najib, Abdeslam Benali

**Affiliations:** 1 Service de Psychiatrie, Hôpital Militaire Avicenne, Marrakech, Maroc; 2 Faculté de Médecine et de Pharmacie, Université Cadi Ayyad, Marrakech, Maroc

**Keywords:** Corticoïdes, culture, effets secondaires psychiatriques, Corticosteroids, culture, psychiatric secondary effects

## Abstract

Les effets secondaires psychiatriques des corticoïdes sont décrits depuis longtemps. Certaines réactions sont sévères et concernent environ 5% des patients. Ces effets secondaires sont plus difficiles à évaluer lorsque le recours aux corticoïdes sort du cadre thérapeutique habituel et s'intègre dans une automédication en lien avec certaines influences culturelles. Nous rapportons le cas d'une jeune femme ayant présenté un épisode psychotique aigu dans les suites d'une auto-prise de corticoïdes dans un but de gain de poids. Nous discutons l'intérêt d'un diagnostique et d'une prise en charge précoce ainsi que l'importance du volet préventif multidisciplinaire, en particulier devant l'implication de l'aspect culturel dans l'observation présentée.

## Abstract (en)

Psychiatric side effects secondary to corticosteroids have been described for a long time. Some reactions are severe, occurring in approximately 5% of patients. These side effects are more difficult to evaluate when corticosteroids are assumed without medical supervision, practicing self-medication influenced by some cultural factors. We here report the case of a young woman with acute corticosteroid-induced psychotic episode. The patient had assumed corticosteroids in an attempt to gain weight. We here highlight the role of diagnostic tests and early management of patients as well as of an effective multidisciplinary strategy, in particular when cultural involvement of patients occurs, as in the case of our patient.

## Introduction

Depuis leur utilisation dans les années 1950, les corticostéroïdes ont vu leur prescription s'élargir dans différentes disciplines médicales. Leurs effets thérapeutiques dans de multiples pathologies sont, cependant, obtenus au prix d'effets secondaires tout aussi variés. À côté des effets indésirables systémiques (métaboliques, endocriniens, dermatologiques, immunologiques etc.), les corticoïdes ont une action pharmacologique cérébrale pouvant intéresser la régulation veille-sommeil, la mémoire et l'humeur [1]. La prévalence des troubles psychiatriques liés aux corticoïdes varie entre 1,8% et 57% en fonction des études [1]. Ils restent sous-estimés car peu dépistés en clinique, et les études les concernant sont peu disponibles dans la littérature [2,3]. L'estimation de cette prévalence s'avère encore plus difficile lorsque la prise des corticoïdes sort de son cadre thérapeutique habituel et s'intègre dans une dimension d'auto médication ne bénéficiant d'aucun suivi ni contrôle médical. Cette auto médication aux corticoïdes concerne une population de femmes de certaines régions du Maroc et se fait essentiellement dans un but de prise de poids. En effet, dans ces régions, la rondeur est considérée comme un critère de beauté féminine et la surcharge pondérale est un phénomène culturellement admis. Malheureusement ce mésusage des médicaments corticoïdes n'est pas sans risques sanitaires parfois sérieux. Nous proposons l'observation d'une jeune femme insatisfaite de son image corporelle et qui, sous l'influence de ses collègues, va avoir recours à la consommation de produits corticoïdes afin de gagner du poids.

## Patient et observation

Mlle I, âgée de 21 ans, célibataire, vit avec sa mère et sa sœur dans une ville du sud marocain. Elle travaille en tant qu'ouvrière et n'a aucun antécédents médicaux ni chirurgicaux ni neuropsychiatriques notoires. Mlle I est admise le matin au service d'accueil des urgences de l'hôpital militaire, amenée par sa famille. La présentation clinique à son arrivée est faite d'une agitation psychomotrice, associée à un syndrome délirant polymorphe, dominé par une thématique de persécution, des hallucinations auditives et un trouble de l'orientation temporo-spaciale. La perturbation de l'humeur se traduit par une élation thymique et une logorrhée alternant avec des propos de menaces auto agressives. Ce tableau clinique a débuté trois jours auparavant par une irritabilité, une anxiété et une insomnie avec quelques éléments délirants. Le diagnostic d'un accès psychotique aigu a été retenu. Les données anamnestiques fournies par la famille nous informent que, depuis une semaine, Mlle I est sous une automédication par un produit proposé par certaines collègues pour « grossir ». Il s'agissait d'un médicament à base de dexaméthasone connu des femmes de la région « pour ses vertus oréxigènes et de gain de poids », et son usage est largement répandu entre elles. Les examens réalisés chez Mlle I comprenant: bilans sanguins, sérologies, une recherche de toxiques dans les urines, une TDM cérébrale, se sont révélés normaux. L'hypothèse de l'origine iatrogène aux corticoïdes de cet accès semblait ainsi très probable. La patiente est hospitalisée dans le service de psychiatrie et a bénéficié d'un traitement antipsychotique à base de risperidone à la posologie progressive jusqu'à 4mg/j associé à un traitement anxiolytique les premiers jours. Ce traitement était bien toléré et a permis un amendement rapide de la symptomatologie psychiatrique au bout d'une semaine. L'antipsychotique a été maintenu, en suivi ambulatoire régulier, à la même posologie pendant six semaines puis réduit à la dose de 2mg/j. Il a été couplé d'une intervention psychothérapeutique et psycho-éducative. L'arrêt du traitement est décidé au bout de six mois. La patiente a pu reprendre son activité professionnelle et les contrôles en consultation jusqu'à 18 mois étaient satisfaisants. Cette présentation clinique illustre le tableau d'un accès psychotique aigu caractérisé. Sa survenue interroge le rôle de la corticothérapie. L'installation rapide après la prise du corticoïde, la résolution précoce des symptômes psychotiques ainsi que l'absence d'antécédents psychiatriques ni de notion d'intoxication par une autre substance sont autant d'éléments pouvant être en faveur d'un lien direct entre la survenue de cet épisode et l'introduction du corticostéroïde.

## Discussion

Les troubles psychiatriques cortico-induits sont très variés et peuvent balayer un champ très large de la pathologie psychiatrique [4]. Insomnie, anxiété, troubles cognitifs, troubles de l'humeur, idées suicidaires, agitation, état confusionnels [2,5,6]. Ces manifestations psychiatriques ont été décrites depuis plus d'une soixantaine d'années par Rome et Braceland [7] et classées en quatre différents stades: 1) le premier se traduit par une certaine euphorie, une baisse du sentiment de fatigue avec une impression de facilité intellectuelle qui peuvent passer inaperçues; 2) au second stade les signes psychiques deviennent plus marqués avec une excitation et une insomnie. Ces deux premiers stades concernent près de deux tiers des patients; 3) le troisième stade correspond à des tableaux psychiatriques caractérisés de troubles anxieux et de variations thymiques franches; 4) le quatrième stade se caractérise par des réactions psychiatriques plus spectaculaires réalisant des épisodes psychotiques aigus ou confusionnels. Ces réactions sévères sont observées en moyenne chez 5,7% des patients [3,5,8]. Le délai d'apparition des manifestations psychiatriques iatrogènes aux corticoïdes est variable. Généralement il est court, en moins d'une semaine [5,8], notamment pour les symptômes maniaques. La symptomatologie dépressive et anxieuse est observée, plus volontiers, lors d'une corticothérapie plus prolongée [2,5,9,10]. Notre observation illustre parfaitement le type de réaction psychiatrique sévère, installée rapidement après introduction du corticostéroïde. Certains facteurs de risques d'apparition de troubles psychiatriques liés à la corticothérapie ont été proposés. La dose du corticoïde administrée semble être le facteur de risque le plus déterminant: le Boston Collaborative Drug Surveillance Program [11] rapporte dans son étude, que les troubles psychiatriques ont été observés chez: a) 1,3% des patients traités par moins de 40 mg/j de prednisone; b) 4,6% des patients recevant entre 40 et 80 mg/j; c) 18,4% des patients recevant plus de 80 mg/j. La dose du corticoïde ne permet, cependant, pas de prédire de la nature de la réaction psychiatrique ni de sa sévérité ou sa durée [8]. Le type de la molécule corticoïde ne semble pas être déterminant [12]. Le sexe féminin parait également un facteur prédisposant [4,5]. Barrami *et al.* [2], dans leur étude, soulignent cette prédominance féminine. Les femmes présenteraient plus des épisodes dépressifs, les hommes des épisodes maniaques [5].

Dans notre observation, il était difficile de préciser la dose du corticoïde ingérée ni, d'ailleurs, les interactions médicamenteuses qui auraient pu se produire dans cette préparation artisanale. La quête intense de gain pondéral rapide décrit chez Mlle I ainsi que la sévérité du trouble présenté laissent supposer le recours à des doses importantes du corticoïde. Le sexe féminin de notre patiente pourrait avoir favorisé l'apparition des symptômes, mais il ne s'agit que d'un seul cas pour en déduire de telle conclusion. De plus, ce phénomène culturel d'automédication par les corticoïdes ne concernait que les femmes et, par conséquent, le facteur sexe ne peut ici être pris en considération. Les mécanismes physiopathologiques de l'apparition d'effets secondaires iatrogènes aux corticoïdes restent mal établis. Certaines hypothèses considères l'effet des corticostéroïdes sur les systèmes dopaminergique et cholinergique centraux, ou suggèrent une baisse de la sécrétion de la sérotonine qui est largement impliquée dans la régulation de l'humeur et le comportement [3,4,9,13,14]. D'autres hypothèses postulent un effet neurotoxique sur l'hippocampe avec réduction de son volume [15]. L'étude de Brown *et al.* [16] portant sur 17 patients retrouve, en effet, une baisse du volume hippocampal chez les patients sous corticothérapie par rapport au groupe contrôle. La prise en charge des troubles psychiatriques cortico-induits n'est pas codifiée. En général elle passe d'abord par la réduction progressive de la posologie du corticoïde jusqu'à une dose minimale efficace, voire l'arrêt complet de la corticothérapie. Cette attitude peut permettre, à elle seule, de faire disparaitre la symptomatologie psychiatrique. Or cette réduction n'est pas toujours possible à cause de la pathologie sous-jacente cortico-traitée, comme elle peut s'avérer insuffisante en raison de l'intensité ou la persistance des troubles. Cela justifie d'une prescription d'un traitement spécifique. Le choix de la molécule varie significativement en fonction des études. Il semble dépendre essentiellement de l'expérience des praticiens, en l'absence de recommandations claires de prise en charge [3,13]. Certaines publications montraient l'efficacité des neuroleptiques classiques type haloperidol ou chlorpromazine surtout pour des symptômes maniaques [17,18]. Parmi les antipsychotiques de seconde génération, l'olanzapine reste la molécule la plus documentée avec une efficacité sur la symptomatologie maniaque ou mixte. La risperidone semble aussi efficace pour des symptômes psychotique comme le délire, les hallucinations mais également des symptômes hypomaniaques [8]. L'amélioration clinique se voit en quelques jours à quelques semaines pour les deux molécules. Le recours à d'autres antipsychotiques tel que la quétiapine, l'aripiprazole a également été rapporté [13].

Par ailleurs, certains auteurs ont proposé l'utilisation de molécules thymorégulatrices comme la lamotrigine, le valproate de sodium ou la phenïtoine [8]. Pour Falk *et al.* [19], la prescription du lithium conjointement pendant la corticothérapie préviendrait l'apparition d'effets secondaires psychiatriques cortico-induits. Concernant les antidépresseurs, il semble que les ISRS, IRSNA: sertraline, fluoxetine, venlafaxine auraient un effet bénéfique sur les symptômes dépressifs [8]. Cette symptomatologie serait, cependant, aggravée sous antidépresseurs tricycliques [20]. Dans le cas clinique que nous avons présenté, la décision d'arrêt du corticoïde ne posait pas de problème puisque cette corticothérapie ne s'intégrait pas dans un cadre thérapeutique. L'intensité du trouble présenté a, toutefois, nécessité le recours à un antipsychotique, la risperidone, associé au départ à un anxiolytique. Cette attitude thérapeutique s'est avérée efficace et a permis un amendement des symptômes en une semaine. Ceci rejoint les résultats décrits dans la littérature. Au-delà des écueils de dépistage et de prise en charge, les troubles psychiatriques iatrogènes aux corticoïdes interrogent une autre dimension aussi importante, celle de la prévention. S'il n'existe pas à ce jour de recommandations claires quant à des mesures préventives particulières, il parait évident que cette prévention pourrait s'appuyer sur une information bien fournie. En effet, les médecins somaticiens doivent toujours, tenir compte de ces effets secondaires psychiatriques et informer leurs patients, cortico-traités, sur les risques de leur survenue ainsi que sur les différents signes cliniques prodromiques. Ceci permettrait un dépistage précoce et par conséquent, une intervention rapide afin d'éviter des réactions plus sévères [20]. Ce rôle des médecins peut être complété par celui des pharmaciens qui peuvent être un acteur intéressant dans cette information du fait de leur contact plus régulier avec les patients et leurs familles [13].

## Conclusion

Les troubles psychiatriques cortico-induits sont très variés et intéressent une proportion très large de patients sous corticothérapie. Certaines réactions sont sévères avec un potentiel de gravité imposant des actions thérapeutiques multidisciplinaires associant psychiatres et somaticiens. L'information des équipes soignantes, des patients et de leurs familles sur la possibilité de ces effets secondaires psychiatriques liés aux corticostéroïdes permettrait un dépistage et une prise en charge précoces et éviterait la survenue de troubles graves. Dans le cas particulier de notre observation, le recours clandestin aux corticoïdes sous tendu par des dimensions culturelles, impose des mesures beaucoup plus sérieuses qui doivent intégrer, outre les professionnels de santé, les travailleurs sociaux ainsi que les médias dans le but d'éclairer la population sur les risques, aussi bien somatiques que psychiatriques de cette automédication. Des enquêtes plus larges sont alors nécessaires pour pouvoir mesurer l'ampleur de ce phénomène culturel et mettre en place des mesures adaptées.

## References

[1] Fietta P, Fietta P (2007). Glucocorticoids and brain functions. Riv Biol.

[2] Barrimi M, Aalouane R, Aarab C, Hafidi H, Baybay H, Soughi M (2013). Corticothérapie prolongée et troubles anxieux et dépressifs: étude longitudinale sur 12 mois. L'Encéphale.

[3] Carle G, Abgrall-Barbry G (2016). Conduites suicidaires et corticothérapie?: à propos d'un cas. L'Encéphale.

[4] Warrington TP, Bostwick JM (2006). Psychiatric adverse effects of corticosteroids. Mayo Clinic Proceedings.

[5] Kenna HA, Poon AW, de los Angeles CP, Koran LM (2011). Psychiatric complications of treatment with corticosteroids: review with case report: corticosteroid psychiatric complications. Psychiatry and Clinical Neurosciences.

[6] Morrow SA, Barr J, Rosehart H, Ulch S (2015). Depression and hypomania symptoms are associated with high dose corticosteroids treatment for MS relapses. Journal of Affective Disorders.

[7] Rome HP, Braceland FJ (1952). The psychological response to ACTH, cortisone, hydrocortisone, and related steroid substances. Am J Psychiatry.

[8] Muzyk AJ, Holt S, Gagliardi JP (2010). Corticosteroid psychosis: stop therapy or add psychotropics'o Off-label antipsychotics, mood stabilizers, and anticonvulsants could help. Current Psychiatry.

[9] Iskandar JW, Wood RL, Ali R, Alemu F (2011). Panic attack induced by a single dose of prednisone. Ann Pharmacother.

[10] Sirois F (2003). Steroid psychosis: a review. Gen Hosp Psychiatry.

[11] Naber D, Sand P, Heigl B (1972). Acute adverse reactions to prednisone in relation to dosage. Clin Pharmacol Ther.

[12] Ricoux A, Guitteny-Collas M, Sauvaget A, Delvot P, Pottier P, Hamidou M (2013). Troubles psychiatriques induits par la corticothérapie orale: mise au point sur la nature, l'incidence, les facteurs de risque et le traitement. La Revue de Médecine Interne.

[13] Kusljic S, Manias E, Gogos A (2016). Corticosteroid-induced psychiatric disturbances: it is time for pharmacists to take notice. Research in Social and Administrative Pharmacy.

[14] Vázquez DM, Neal CR, Patel PD, Kaciroti N, López JF (2012). Regulation of corticoid and serotonin receptor brain system following early life exposure of glucocorticoids: long term implications for the neurobiology of mood. Psychoneuroendocrinology.

[15] Preda A, Fazeli A, McKay BG, Bowers MB, Mazure CM (1999). Lamotrigine as prophylaxis against steroid-induced mania. J Clin Psychiatry.

[16] Brown ES, Woolston DJ, Frol A, Bobadilla L, Khan DA, Hanczyc M (2004). Hippocampal volume, spectroscopy, cognition, and mood in patients receiving corticosteroid therapy. Biol Psychiatry.

[17] Wada K, Yamada N, Sato T, Suzuki H, Miki M, Lee Y (2001). Corticosteroid-Induced Psychotic and Mood Disorders: Diagnosis Defined by DSM-IV and Clinical Pictures. Psychosomatics.

[18] Bloch M, Gur E, Shalev A (1994). Chlorpromazine prophylaxis of steroid-induced psychosis. Gen Hosp Psychiatry.

[19] Falk WE, Mahnke MW, Poskanzer DC (1979). Lithium prophylaxis of corticotropin-induced psychosis. JAMA.

[20] Airagnes G, Rouge-Maillart C, Garre J-B, Gohier B (2012). Homicide et épisode psychotique aigu cortico-induit?: à propos d'un cas. L'Encéphale.

